# Influenza A Virus Infection in Cats and Dogs: A Literature Review in the Light of the “One Health” Concept

**DOI:** 10.3389/fpubh.2020.00083

**Published:** 2020-03-20

**Authors:** Stéphanie Borland, Patrice Gracieux, Matthew Jones, François Mallet, Javier Yugueros-Marcos

**Affiliations:** ^1^bioMérieux S.A./BioFire Diagnostics LLC Research and Development, Centre Christophe Mérieux, Grenoble, France; ^2^BioFire Diagnostics LLC, Salt Lake City, UT, United States; ^3^Joint Research Unit, Hospice Civils de Lyon, bioMérieux S.A., Centre Hospitalier Lyon Sud, Pierre-Benite, France

**Keywords:** influenza, one health, zoonosis, dog, cat, interspecies transmission, public health

## Abstract

Influenza A viruses are amongst the most challenging viruses that threaten both human and animal health. Constantly evolving and crossing species barrier, the emergence of novel zoonotic pathogens is one of the greatest challenges to global health security. During the last decade, considerable attention has been paid to influenza virus infections in dogs, as two canine H3N8 and H3N2 subtypes caused several outbreaks through the United States and Southern Asia, becoming endemic. Cats, even though less documented in the literature, still appear to be susceptible to many avian influenza infections. While influenza epidemics pose a threat to canine and feline health, the risks to humans are largely unknown. Here, we review most recent knowledge of the epidemiology of influenza A viruses in dogs and cats, existing evidences for the abilities of these species to host, sustain intraspecific transmission, and generate novel flu A lineages through genomic reassortment. Such enhanced understanding suggests a need to reinforce surveillance of the role played by companion animals-human interface, in light of the “One Health” concept and the potential emergence of novel zoonotic viruses.

## Introduction

Influenza is an acute infectious respiratory disease caused, in humans, by influenza type A or type B viruses. While the latter type circulates only among humans, influenza A viruses (IAV) can also be isolated from a wide variety of animal species. Wild migratory birds and bats are main natural reservoirs, from where virus uses to spill over into other animal hosts like ducks, chickens, horses, pigs, whales, cats, dogs, etc. IAVs viruses commonly exhibit restricted host range, but occasionally transmit from one species to another host (1). Notably, numerous spillover events arose primarily from poultry and swine that pose a significant threat to human health as historically, most human pandemics have emerged from avian and swine hosts (2, 3). In a world where the number of cat and dog owners is increasing, and social behavior tends to enroll these animal species as family members (4–6), this review aims at providing an up-to-date picture of the epidemiology of IAV in dogs and cats and their transmission modes. Their evolution and the consequences of genetic reassortments of IAV are further discussed, leading us to provide recommendations on surveillance tools and on the role that diagnostic tools could play in the “One Health” concept approach.

## Epidemiology of IAV in Cats and Dogs

**Figure 1:**
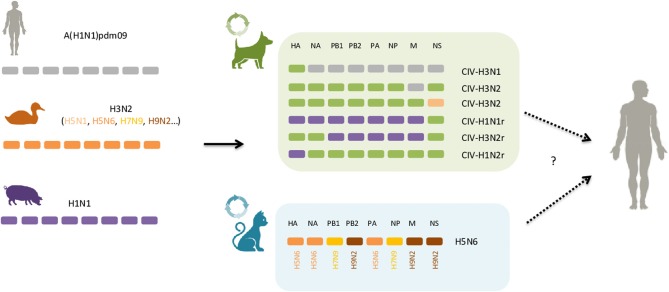
Dogs and cats as mixing vessels for influenza A virus. In green and light blue boxes are represented the genomic structure of reassortants that have been reported in dogs and cats, respectively. The host origin of the eight segments of viral RNA are displayed as follows: in gray, human; orange, avian; purple, swine; green, canine. Solid arrows indicate well-described interspecies events, circle arrows indicate gene reassortment events and dashed arrows represent the potential for those novel viral combinations to jump to humans, although no such case has been reported so far. See text for further details.

Currently, five subtypes of IAV are frequently described in the literature as cause of acute respiratory illness in cats and dogs: H3N8, H3N2, low pathogenic avian influenza virus (LPAIV) H7N2, high pathogenic avian influenza virus (HPAIV) H5N1 as well as the pandemic H1N1 virus, circulating nowadays as the seasonal flu virus in humans (Table 1). Other subtypes, mainly from avian origin, or a result of genetic reassortments following co-infections of different avian, swine and human IAV, have also been isolated from cats and dogs with respiratory disease (i.e., H5N6, H5N2, H3N1) (Figure 1). Reports of infection with human influenza A seasonal types have been published, but to a much lesser extent, not being covered by this review.

**Table 1:** Overview of major natural influenza A subtype infections and reassortment events reported in dogs and cats.

**Influenza A subtype^a^**	**First isolation (localization and year)**	**Host species (origin)**	**Currently reported geographic distribution^b^**	**Severity of the disease^c^**	**Intraspecies transmissibility^d^**	**Transmission to humans^e^**	**References**
CIV-H3N8	Florida, USA, 2004	Dogs (Horse)	USA^b^, UK, Canada	+	+	Never reported	(7–9)
CIV-H3N2	China, 2006	Dogs and Cats (Avian)	Southeast Asia^b^, North America^b^	++	++	Never reported	(10–16)
LPAIV H7N2	New York City, USA, 2016	Cats (Avian)	USA	+	+	Reported once	(17, 18)
HPAIV H5N1	Thailand, 2006	Dogs and Cats (Avian)	Thailand, China, Austria, Germany	+ + +	–/+	Never reported	(19–25)
A(H1N1)pdm09	Italy, 2009	Dogs and Cats (Human)	USA, China, Mexico, Italy	+ + +	+	Reverse zoonosis	(26–34)
CIV-H3N1*	South Korea, 2010	Dogs (Human)	Unknown	-	-	Never reported	(35)
CIV-H3N2*	South Korea, 2012	Dogs (Human)	Unknown	++	+	Never reported	(36)
CIV-H1N1r*	China, 2015	Dogs (Swine)	China	++	Unknown	Never reported	(37)
CIV-H1N2r*	China, 2014	Dogs (Swine)	China	+	Unknown	Never reported	(37)
CIV-H3N2r*	China, 2015	Dogs (Swine)	China	+	Unknown	Never reported	(37)

a *CIV, Canine Influenza Virus; HPAIV, High Pathogenic Avian Influenza Virus; pdm, pandemic; LPAIV, Low Pathogenic Avian Influenza Virus; r, reassortant and further highlighted by an asterisk*.

b *Refers to endemic subtype in canine population*.

c *Severity was defined based one the following criteria: + mild respiratory symptoms; ++ severe respiratory symptoms; + + + systemic infection*.

d *Intraspecies transmissibilty refers to dog-to-dog and cat-to-cat transmission events and was classified as follows: - no evidence of case-to-case transmission; –/+ limited transmission; ++ efficient spreading*.

e *Reverse Zoonosis refers to an influenza subtype that can be transmitted from humans to companion animals*.

Canine influenza virus (CIV) H3N8 subtype was reported in 2004 in an outbreak of severe respiratory disease in racing greyhounds, although serological evidence suggested it emerged around 1999 (7, 38, 39). The virus has since been considered endemic in the canine population in the US, affecting both racing greyhounds and pets (38). Affected dogs exhibited various degrees of pneumonia and CIV-H3N8 spread readily from infected dogs to other susceptible dogs through direct contact (40). Other spillover CIV-H3N8 infections have been sporadically reported in other parts of the world, such as in Canada, UK, Australia, China and Nigeria (41), but no evidence of continuing circulation of CIV-H3N8 has been reported in these areas, and the risk of emergence appears very low (8, 41–45). The CIV-H3N8 subtype appeared to have been primarily maintained in large urban animal shelters, where susceptible animals live in dense population (46–50). Noteworthy, no CIV-H3N8 infection has been reported since 2016.

Around 2006, a novel H3N2 subtype arose in dogs in China and South Korea, and then rapidly spread into several areas of Southeast Asia, where it is now stably circulating in the canine population becoming endemic (and thus referred to as CIV-H3N2) (10–12, 51). CIV-H3N2 was first identified in the US in 2015, as a causative agent of epidemic outbreaks of severe respiratory disease that affected more than 1,000 dogs in Chicago and nearby areas (13, 52, 53). Its spread within the US and Canada may have arisen from rehoming of dogs rescued from meat markets imported from Asia to US (14). Despite local control measures, the virus has continued circulating among the canine population and has spread to several other areas of the country, indicating sustained dog-to-dog transmission in the US, through combinations of multiple incursions into the US from Asia and a series of localized outbreaks and fade-outs (13, 53). Some interspecies transmission events to cats have also been reported, being further described in section Influenza A Virus Interspecies Transmission to Cats and Dogs of this article (15).

A LPAIV of the H7N2 subtype, which circulated in live poultry markets in the eastern and northeastern US during 1994–2006, was identified as the causative source of an outbreak in a cat shelter in New York City in December 2016, subsequently spreading to multiple shelters in the states of New York and Pennsylvania. Infected cats experienced clinical signs of coughing, sneezing, and runny nose from which they fully recovered (17). Under experimental conditions, feline H7N2 subtype was found to replicate efficiently in cat upper and lower respiratory tracts and had the ability to transmit among cats, indicating adaptation of this avian H7N2 to felines. During the outbreak, a veterinarian who treated the animals also became infected with feline H7N2 influenza virus and experienced respiratory symptoms (17). In addition, another case of cat-to-human transmission was reported in an animal shelter employee who experienced mild illness symptoms and who had direct exposure to ill cats. No evidence of human-to-human transmission has been reported so far (54, 55).

HPAIV H5N1 subtype initially originated in China in 1996 and it has spread since then into many areas in the world, causing infections in birds of many species (56). Dogs and cats have been infected by direct contact with affected birds, especially by eating raw poultry (19–21). Of particular concern, severe symptoms, not limited to respiratory organs but also hepatic and gastrointestinal were reported, and in many cases systemic infection was evidenced. Subclinical infection of cats with H5N1 was also reported after contact with infected birds or their excrement (22), thus indicating that cats may serve as a potential asymptomatic H5N1 reservoir. Nevertheless, low prevalence of H5N1 antibodies was reported in cats sera, even in areas in which birds infected with HPAIV H5N1 had been documented (23, 57, 58).

In Italy, 2009, a pandemic H1N1 [A(H1N1)pdm09] outbreak occurred in a colony of 90 caged stray cats (26). Half of the animal colony had signs of severe acute respiratory and gastrointestinal infections. Serum samples and pharyngeal swabs were collected from 38 of the 65 surviving cats, and more than half (55%) of the tested cats were seropositive for the presence of A(H1N1)pdm09 antibodies and two swabs were positive for the presence of A(H1N1)pdm09 by PCR, highlighting cat-to-cat transmission of the virus (26). Furthermore, several sporadic cases of natural infections with A(H1N1)pdm09 influenza viruses were also reported in domestic cats exhibiting clinical signs of acute respiratory infection (27–29, 59, 60). In the latter cases, the most likely source of infection was found to be people in the household. Indeed, as owners of infected cats also had a history of severe respiratory disease, with prior infection with A(H1N1)pdm09 virus confirmed (59) or coinciding with periods of increased influenza activity (28). More, seroprevalence of antibodies against A(H1N1)pdm09 was three times more prevalent in pet cats than free-roaming cats (61). In dogs, some rare cases of natural A(H1N1)pdm09 infections have also been documented so far (30), notwithstanding the ability of the virus to replicate in dogs respiratory tract in an experimental setting, even though symptoms were very mild, and appeared to rather transmit inefficiently from dog-to-dog (30).

## Influenza a Virus Interspecies Transmission to Cats and Dogs

Successful interspecies transmission of IAV is dependent on both host and virus genetic factors, and subsequent spread within the new host population requires a period of adaptation of the virus to the new host (1, 62). Critical host and viral determinants involved in virus specificity and further mechanisms in the adaptation to cats and dogs are described.

### Avian Interspecies Transmission

Among those determinants in the specificity of IAV for a host, we would highlight the presence of virus receptors on susceptible host cells, especially those to which the viral hemagglutinin (HA) is able to bind. Most avian and human IAVs have preference for specific receptor types having glycans with sialic acid residues in α-2,3 (avian receptor) or in α-2,6 (mammalian receptor) linkages (63). Because canine and feline upper and lower respiratory tract epithelium display α-2,3 sialic acid receptors, direct transmission of avian influenza subtypes from poultry to dogs or cats is possible (11, 64). Transmission mechanisms have been mostly documented for CIV-H3N2 infection, even though up-to-date, other natural infections with avian IAV types have been reported in China: H9N2 (65, 66), H5N1, H5N6 (67), H5N2 (68).

In most canine H3N2 cases reported, genetic analysis showed that all genes of the isolates were closely related to an avian H3N2 IAV, suggesting that the entire genome of the avian influenza virus had been transmitted to dogs with no sign of gene reassortment (10, 11, 69). The virus was found to be most widespread in kennels and in meat dog farms, likely due to the close physical contact between infected poultry and dogs in these host-dense environments (16, 69, 70). From a molecular stand point, most H3N2 canine isolates were found to have at least two mutations in the HA proteins (Ser159Asn and Trp222Leu) that may have facilitated H3N2 influenza A virus jump from birds to dogs (52, 71). Furthermore, it is likely that gradual accumulation of mutations throughout the eight gene segments may have resulted in specific adaptation of H3N2 into canids (69, 72), although the evolutionary rate of the NA segment was higher than the seven others (69). This is further supported by the fact that (2012–2013) Korean canine H3N2 isolates replicated at higher titer and induced more severe clinical symptoms than 2009 isolates, clearly indicating that the canine H3N2 virus is continuously evolving in the canine population.

Noteworthy, CIV-H3N2 further acquired the ability to naturally infect cats, as first reported in a Korean animal shelter (15) (Table 1). Genomic sequence analysis of H3N2 feline isolate displayed high sequence similarities (98.0–99.8%) with canine H3N2 isolate, thus suggesting that CIV-H3N2 can be naturally transmitted from dogs to cats without prior adaptation (15, 73, 74). Hence, this indicates that cats may play an intermediate host role in transmitting the H3N2 virus among feline and canine species.

### Equine Interspecies Transmission

Canine H3N8 originated after the transfer of an equine influenza virus (EIV), likely through close contact with infected horses (7). Phylogenetic analysis of the HA H3N8 viral genomic sequences from horses and dogs highlighted that all canine H3N8 sequences clustered together in a single monophyletic group, distinct from EIV (7). So far, no evidence of reassortment with other subtypes has been reported. A comparison of equine and canine H3N8 sequences highlighted key amino acid residues that may be involved in receptor binding specificity and host cell tropism (38, 75, 76). Interestingly, as for H3N2, structural, and receptor binding analyses support the role of the HA Trp222Leu mutation in facilitating viral interspecies transmission from equine to canine (77–79). However, CIV-H3N8 has not been found to be phenotypically different from equine H3N8 strains in terms of replicability and infectivity, thus suggesting that cross-species transmission and adaptation of influenza viruses may be rather mediated by subtle changes in virus biology (76, 80). Furthermore, recent analyses of the amino acid sequence from emerging and contemporary CIV-H3N8 isolates, revealed that significant antigenic drift may have occurred. Altogether since its introduction into the canine population, evolution dynamics studies of CIV-H3N8 suggested that it evolved and diverged into multiple lineages (81).

### Human Interspecies Transmission

To date, serological evidence suggest that cats and dogs could be infected worldwide with human seasonal A(H1N1)pdm09 and H3N2 strains probably by direct transmission from their owners (31, 32, 82, 83). Several points support this hypothesis: (i) in most cases reported so far, animal caretakers or owners had themselves history of flu-like illness and for some of them confirmed by PCR; (ii) susceptibility of cats and dogs correlated well with influenza prevalence in the human population and even followed a seasonality pattern as in humans, and (iii) virus isolation and sequence analysis of all eight genes of the canine isolates showed high nucleotide similarity thus suggesting that human viruses could therefore jump into dogs and cats, without prior adaptation. However, details about molecular determinants potentially related to transmission have not been unraveled so far.

## Subtypes Arose From Genetic Reassortment

Major attention has traditionally focused on swine as key mammalian “mixing vessels” hosts for the reassortment of influenza viruses from different host species (84). As both α-2,3 sialic acid receptors and α-2,6 receptors residues are distributed throughout its respiratory tract, swine serve as a vehicle for influenza virus genetic reassortment, allowing avian, swine, and human IAVs subtypes to reassort following co-infection. Knowing that both receptors have been found in dog and cat respiratory organs, as revealed by lectin histochemistry analyses (37, 85, 86), dogs and cats could be dually or sequentially infected with avian- and/or mammalian influenza viruses, making them possible hosts for the generation of a viruses with novel genome combinations, with epidemic and/or pandemic potentials.

Different genetic reassorted subtypes have arose in dogs (Table 1, Figure 1). During a surveillance effort in 2012 in South Korea, a novel strain of the H3N1 subtype was isolated from canines, and whole-genome sequencing showed that it contained the HA gene segment from CIV-H3N2 and the remaining seven other gene segments from human A(H1N1)pdm09 (35). Since then, at least four other reassortants involving CIV-H3N2 and A(H1N1)pdm09 have been isolated from dogs in Southern Asia, including a CIV-H3N2 harboring the M gene segment of human A(H1N1)pdm09 (36, 87). The emergence of these novel CIV-H3N2 reassortants likely arose through co-infection of CIV-H3N2 and A(H1N1)pdm09 virus, correlating with high co-positivity for both canine H3N2 and A(H1N1)pdm09 antibodies in the canine population (11, 35). Remarkably, multiple genomic reassortment between swine-origin subtypes of the H1N1 and endemic canine H3N2 lineages co-circulating in dogs were recently reported in pet dogs in China (Table 1) (88). Moreover, in a large-scale analysis of sequence data of IAVs from various species, the NS gene of a CIV-H3N2 subtype isolated from a Chinese dog in 2007 was found to be closely related to H5N1 avian influenza viruses, indicating that reassortment may also have occurred between canine H3N2 and avian H5N1 (89). Of particular concern, some CIV-H3N2 reassortants demonstrated the ability to infect and efficiently transmit to cohoused dogs in experimental settings, thus supporting potential adaptation of novel subtypes to canine populations (36, 80).

In cats no reassortment between avian and mammalian IAVs was thought to occur until recently, where a novel reassortant of the H5N6-subtype influenza viruses was isolated from two cats in eastern China (90). Both viruses were sequenced and genetic analysis showed that these viruses received their genes from three avian subtypes, including H5N6 (HA, NA, PA), H9N2 (PB2, M, NS), and H7N9 (PB1, NP) influenza subtypes viruses isolated in China (Figure 1). Analysis of the receptor-binding preference of the feline isolated H5N6 virus revealed that it possesses both avian- and human receptor specificities. Furthermore, the H5N6 virus was able to replicate to high titer in the lungs of infected in mice without prior adaptation, though it was not lethal, indicating mammalian host adaptation (90).

## Discussion

Evidences exist that companion dogs and cats can have a dual role as influenza A virus hosts, by (i) sustaining inter- and intraspecific transmission and (ii) generating novel IAV through recombination. Although most cases of natural cross-species infections have resulted in limited onward transmission in dogs and cats, two influenza subtypes are now continuing to circulate in dogs (CIV-H3N8 and CIV-H3N2). While the role of cats is less clear and less documented, they still appear to be susceptible mainly to avian influenza infections, even though most of them seem to be rather subclinical (reservoirs). This should be a cause of concern, especially for feral and free-roaming cats that tend to have less controls and closer contact with birds and other farm animals. However, it can be also assumed that because of the feline social organization that prevents direct cat-to-cat contact required for viral transmission, the virus may transmits very inefficiently among feline population.

Moreover, several lines of evidence suggest that dogs and cats should be considered as mixing vessels for the reassortment of novel influenza viruses. Notably, canine influenza viruses, and more particularly those of the CIV-H3N2 subtype, have reassorted multiple times with avian- and mammalian- adapted influenza viruses since their time of emergence, clearly showing that the gene pool of avian, human, and canine viruses is indeed compatible. These new viruses could further spread widely among household dogs and cats and may therefore represent a threat for human health. Up to date only one case of interspecific cat-to-human spill-over has been reported, and this occurred after prolonged and unprotected exposure to ill cats and their respiratory secretions, which indicates that risk for cat-to-human transmission is low (91). Rather infected humans may be the source of pet infection, and the combination of reverse zoonosis (from humans to pets), potential co-infections and gene reassortment may provide a favorable ecosystem for crossing species barrier between pets and humans.

In light of reported epidemiological evidences and current knowledge of the molecular mechanisms behind interspecies transmission and genetic reassortment, it seems of significant importance to enhance active surveillance of cats and dogs under the framework of “One World, One Health,” warranting control and prevention of IAV infections as the threat of an influenza pandemic is a concern. Notably, implementing large scale programs of IAV antibody serosurveillance in canine and feline populations may serve as sentinels for monitoring the overall risk of human exposure to emerging zoonotic influenza viruses. Moreover, information on influenza viruses circulating in canine and feline populations is also crucial for the selection of viruses for effective vaccination programs targeting high risks dog populations (92) and will undoubtedly aid in the prevention and control of future epidemics. The advent of rapid molecular diagnostic tests such as real-time PCR and unbiased next generation sequencing that can directly detect viral pathogens and combined with specific subtyping (i.e., H3N8 and H3N2 in dogs; H1N1 and H7N2 in cats) should also provide earlier warning and enable a more appropriate outbreak control in case of respiratory illness symptoms in cats and dogs. As the genesis of these emerging viruses is not well-understood, further research aiming at investigating the ecology, evolution and mechanisms of IAV at the human–animal interface will help to better understand which virus pose a serious threat to humans.

## Author Contributions

SB performed the review, collected data form literature, and wrote the manuscript. JY-M conceived the idea of the review and helped to revise the manuscript. PG, MJ, and FM revised and provided first feedback for the manuscript. All the authors contributed to manuscript revision, read, and approved the submitted version.

### Conflict of Interest

SB, PG, JY-M, and FM are employees of bioMérieux S.A. MJ is an employee of BioFire Diagnostics LLC.
